# Supplementary material for: Plasmonic ommatidia for lensless compound-eye vision
Source: Nat Commun. 2020 Apr 2;11:1637. doi: 10.1038/s41467-020-15460-0 (PMC7118074; doi:10.1038/s41467-020-15460-0)
Supplement: Supplementary file 1 — Supplementary Information [file 41467_2020_15460_MOESM1_ESM.pdf]

Supplementary Information

Plasmonic ommatidia for lensless compound-eye vision

Kogos et al.

Supplementary Note 1. Grating coupler design

The geometrical parameters of the nanoparticle (NP) arrays in the grating couplers were optimized through extensive two-dimensional finite difference time domain (FDTD) simulations, where the optical transmission through the entire metasurface is computed as a function of polar angle of incidence on the *x*-*z* plane (the plane perpendicular to the long axes of the NPs, as defined in Fig. 2 of the main text) for *p*-polarized light. First, the array period Λ is selected to produce the desired angle of peak detection θ_p according to the Bragg condition discussed in the main text. Next, the NP width (w) and the number of NPs in the array (N) are optimized iteratively, so as to maximize the peak metasurface transmittance at θ_p (T_p) while keeping the background as small as possible. Representative results produced in the course of these simulations are shown in Supplementary Fig. 1. The optimal number of NPs (i.e., the optimal length of the array between the slits and the grating reflector, equal to $N\Lambda$) is determined by the following tradeoff. If N is too small, an exceedingly large fraction of the sample area is occupied by the slits and grating reflector, where light incident at the desired detection angle is not preferentially coupled to surface plasmon polaritons (SPPs) that can then be scattered by the slits into the substrate. If N is too large, a large fraction of the SPPs created by incident light at θ_p (the ones produced furthest away from the slits) are absorbed in the metal film or scattered back into radiation before reaching the slits. Supplementary Fig. 1(a) illustrates the resulting dependence of T_p on N for the metasurface design with θ_p = 30°; the optimal number of NPs in this device is 16.

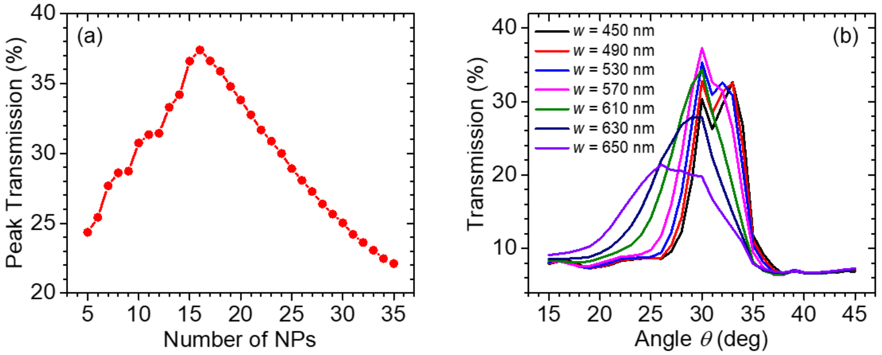

Supplementary Figure 1. Grating-coupler design simulation results for the θ_p=30° device. (a) Metasurface transmission coefficient for 1550-nm *p*-polarized light incident at 30° versus number of NPs in the array. (b) Angle-dependent transmission coefficient for different values of the NP width. In both panels, all other design parameters are set equal to their optimal values presented below.

The NP width w controls the efficiency with which the array can diffract incident light at θ_p into SPPs. In this respect, a reasonable first choice is $w \sim \Lambda/2$, with the exact value depending

on the SPP absorption losses in the metal film. Additionally, another important consideration is provided by the spectral position of the localized surface plasmon resonances (LSPRs) supported by the individual NPs, which strongly depends on w. For the NP dimensions considered in this work, the fundamental (dipolar) LSPR falls in the near-infrared spectral region, as indicated by additional FDTD simulations (not shown). If this resonance overlaps with the design wavelength $\lambda_0 = 1550$ nm, the device angular response at λ_0 is affected in two ways. First, some of the incident light can be absorbed through the excitation and subsequent nonradiative decay of LSPRs, causing a reduction in peak transmission T_p. Second, the SPP-LSPR coupling can significantly modify the SPP dispersion curve [1, 2], leading to a change in the SPP wavelength λ_{SPP} at λ_0, and therefore in the angle of peak detection θ_p determined by the diffraction condition. As an additional complication, for p-polarized light incident at sufficiently large angles, the in-plane and out-of-plane components of the electric field can couple to two distinct SPP-LSPR mixed resonances, leading to two different values of θ_p and therefore two peaks in the device angular response (a similar behavior has been observed in reflection spectroscopy measurements in ref. 2). All the effects just described are illustrated in Supplementary Fig. 1(b), where we plot the angular response of the θ_p=30° device computed for different values of w. The optimal NP width (in this case 570 nm for a period Λ = 992 nm) is selected so as to maximize T_p while at the same time avoiding the appearance of a second peak.

Supplementary Note 2. Grating reflector design

As illustrated in Fig. 2(b) of main text, the grating reflector is a periodic array of rectangular NPs designed to scatter the incoming SPPs (propagating along the $+x$ direction) into light radiating away from the sample along the surface normal, via negative-first-order ($q = -1$) diffraction. Therefore, the array period Λ_{gr} = 1524 nm is selected to match its SPP wavelength at λ_0. Additionally, its unit cells are designed so that all other orders of diffraction (including $q = 0$ and $q = +1$) are suppressed, in order to avoid interpixel crosstalk and spurious signals caused by SPPs propagating towards a neighboring pixel in a photodetector array. This goal can be achieved using a gradient metasurface (GMS) geometry where the reflection phase varies linearly with position along the x direction with negative slope $-2\pi/\Lambda_{gr}$ [3, 4] (functionally equivalent to a blazed diffraction grating of period Λ_{gr}).

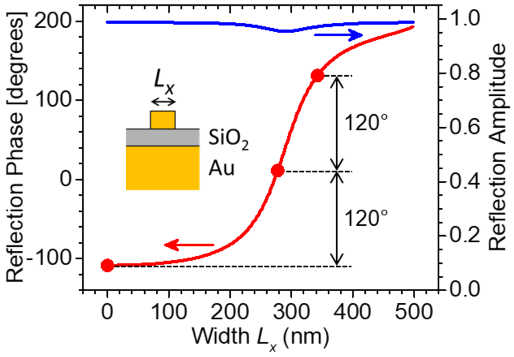

Supplementary Figure 2. GMS design. Reflection phase (red trace) and amplitude (blue trace) of the GMS building block shown in the inset, plotted as a function of NP width L_x for normally incident x-polarized light at λ_0 = 1550 nm. Both traces were computed via FDTD simulations for a periodic (non-diffracting) array of identical NPs with 550-nm period. All layer thicknesses are the same as in the optimized structures described below. The red circles indicate three NP widths providing equally spaced reflection-phase values differing by $2\pi/3$.

Specifically, we follow the approach of ref. 5, where a GMS consisting of rectangular NPs on a metal ground plane was developed to demonstrate unidirectional excitation of SPPs by normally incident light. In this approach, a discretized version of the desired linear phase gradient is obtained by building each unit cell with a small number of different NPs, equally spaced along the x direction and providing equally spaced reflection-phase values across the full 2π range. In the geometry under study, the reflection phase of each NP can be tuned across a large fraction of the entire 2π phase space (while at the same time maintaining a high reflection amplitude > 95 %) by varying its width L_x, as shown by the FDTD simulation results of Supplementary Fig. 2. The dots in the same figure indicate a suitable set of 3 NP widths with equally spaced reflection-phase values differing by $2\pi/3$ (so as to collectively sample the full 2π range). It should be noted that the narrowest NP in this set has zero width (equivalent to a missing NP), which is a particularly convenient choice from a fabrication standpoint. The desired linear phase profile with negative slope $-2\pi/\Lambda_{gr}$ can then be implemented with the periodic repetition of these 3 NPs, equally spaced at a distance of $\Lambda_{gr}/3$ from one another and with progressively decreasing width (i.e., decreasing reflection phase) along the $+x$ direction.

To illustrate the effectiveness of the GMS design, in Supplementary Fig. 3 we compare two otherwise identical device structures providing peak detection at θ_p = +30° with different grating reflectors. The structure of panel (a) is based on the GMS design just described. In the structure of panel (b), the GMS is replaced by a symmetric NP array having the same periodicity (1524 nm) and the same number of repeat units (5), but only one NP in each unit (with 570-nm

width). The traces plotted in the figures are the p-polarized power transmission coefficients of both devices versus polar angle of incidence θ on the x-z plane. In panel (b), in addition to the large peak at the target detection angle θ_p = +30°, a smaller peak at the opposite angle –30° is also observed, caused by SPPs excited in the grating coupler and transmitted across the reflector [here, as in all 2D simulations presented in this work, we use periodic boundary conditions on the lateral boundaries of the simulation window to model multiple repetitions of the entire metasurface structure, as in the actual experimental samples; therefore, each grating reflector is immediately followed by the slits of the next repetition]. In panel (a), this additional undesired peak is almost completely suppressed through the use of the GMS reflector. This comparison clearly illustrates the improved ability of the GMS to efficiently scatter incoming SPPs into radiation, related to the suppression of zero-order diffraction (i.e., straight SPP transmission) by its linear phase gradient.

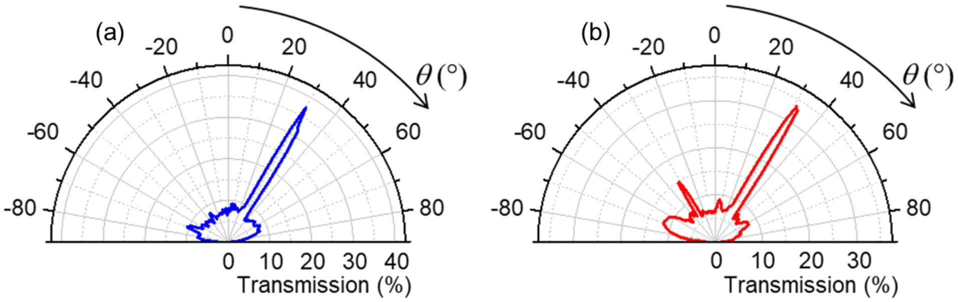

Supplementary Figure 3. Grating reflector design. Calculated p-polarized transmission coefficient at λ_0 = 1550 nm versus angle of incidence θ on the x-z plane, for two otherwise identical metasurfaces with different grating reflectors, consisting of a GMS (a) and a symmetric NP array (b).

Supplementary Note 3. Optimized structures

In the following we describe the geometrical parameters of the optimized device structures developed in this work. Several parameters are common to all structures, selected based on initial FDTD simulations as well as practical considerations related to the available fabrication processes. In all devices, both SiO_2 layers have a thickness of 60 nm, the metal film consists of 5 nm of Ti and 100 nm of Au, and each NP consists of 5 nm of Ti and 50 nm of Au. Each slit section contains 5 slits with 200-nm width and 400-nm center-to-center spacing. Each grating reflector consists of 5 repeat units of 3 equally spaced NPs of different widths (340, 313, and 0 nm) with a period of 1524 nm. It should be noted here that the 313-nm width of the middle NP is somewhat larger than the value suggested by the simulation results of Supplementary Fig. 2. It was found that this deviation produces a smaller background in the angular response, particularly for large negative

angles of incidence (which could not be diffracted away from the grating reflector, and would instead be coupled to surface waves, if all orders of diffraction except for $q = -1$ are strictly suppressed). Additionally, in all devices the overall length along the *x* direction occupied by a slit section, grating coupler, and grating reflector is an integral multiple of the coupler period Λ (the smallest integer that allows accommodating all the desired building blocks). The positions of the slits and grating-reflector NPs within their respective allocated spaces were also optimized to maximize the metasurface peak transmittance T_p.

The remaining key geometrical parameters that determine the angular response of the individual devices are the period Λ, the NP width *w*, and the number of NPs *N* in the grating coupler. Their values were determined with the optimization procedure described above, and are listed in Supplementary Table 1. Finally, in the device designed for peak detection at $\theta_p = 0°$ the grating reflector is absent and replaced by a slit section, since the desired angular response is symmetric.

**Peak angle θ_p [°]**	**Period Λ [nm]**	**NP width *w* [nm]**	**Number of NPs *N***
0	1465	250	15
15	1185	570	18
30	992	570	16
45	879	570	20
60	781	270	29
75	745	270	29

Supplementary Table 1. Key geometrical parameters of the six device structures used in all numerical simulations presented in this work.

Supplementary Note 4. Additional simulated angular response patterns

As discussed in the main text, the devices developed in this work exhibit a strong polarization dependence. In Supplementary Fig. 4(a) we plot the *s*-polarized transmission coefficient of the six optimized metasurfaces of Supplementary Table 1, computed as a function of polar angle of incidence θ on the *x*-*z* plane. All traces shown in this figure are relatively isotropic, with transmission values less than 0.2 % at all angles – i.e., two orders of magnitude smaller than the peak transmission coefficients of the same devices for *p*-polarized incident light [see Fig. 2(c) of the main text]. The same behavior applies for all other azimuthal illumination angles, with the *xz*-

polarized response consistently stronger than the yz-polarized one. To illustrate, in Supplementary Figs. 4(b) and 4(c) we show the calculated far-field radiation patterns through the metasurface of the θ_p=15° device, produced by a dipole located in the Ge substrate and oriented along the x and y directions, respectively (plotted on the same scale). By reciprocity, these patterns are proportional to the x- and y-polarized local field intensities produced at the dipole location by a plane wave incident from the air above, as a function of both illumination angles θ and ϕ. The much larger peak magnitude of the x-polarized pattern in these plots is consistent with the polarization properties described above. In passing, we note that the calculated radiation patterns for z-oriented dipoles are also smaller than those of the x-oriented ones.

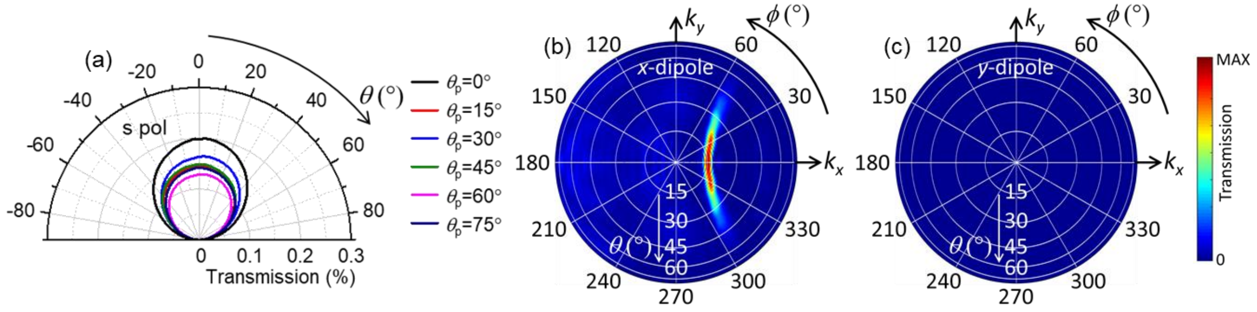

Supplementary Figure 4. Polarization properties of the optimized structures designed in this work. (a) s-polarized transmission coefficient of all six metasurfaces of Supplementary Table 1 versus angle of incidence on the x-z plane. (b) Far-field radiation pattern through the metasurface of the θ_p=15° device, produced by an x-oriented dipole located in the device substrate. (c) Same as (b) for a y-oriented dipole. For a direct comparison, the patterns of (b) and (c) are plotted on the same color scale, which is normalized to the maximum (MAX) transmission value of (b).

Supplementary Figure 5 shows the calculated angular response patterns of the optimized structures designed for peak detection at 15°, 45°, and 75°, computed with the reciprocity-based method just described and summed over all dipole orientations. These plots, together with the maps of Figs. 2(d)-(f), have been used in the image-reconstruction simulations described below.

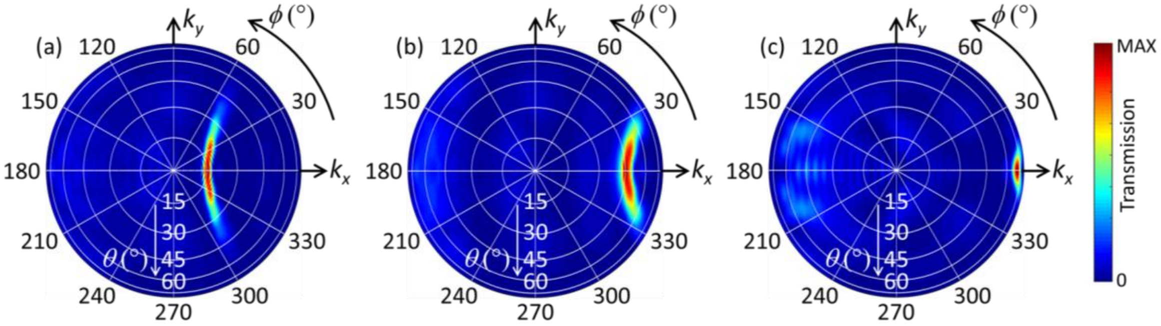

Supplementary Figure 5. Additional simulation results. Transmission coefficient of the metasurfaces designed for peak detection at 15° (a), 45° (b), and 75° (c), summed over all polarizations and plotted as a function of polar and azimuthal illumination angles. In each map, the color scale ranges from 0 to the peak transmission value (MAX) of the corresponding device, to more clearly illustrate the device angular response. The incident light wavelength is 1550 nm.

Supplementary Note 5. Additional measured angular response patterns

The experimental angular response patterns were measured with the setup shown schematically in Supplementary Fig. 6 (described in the Methods section of the main text).

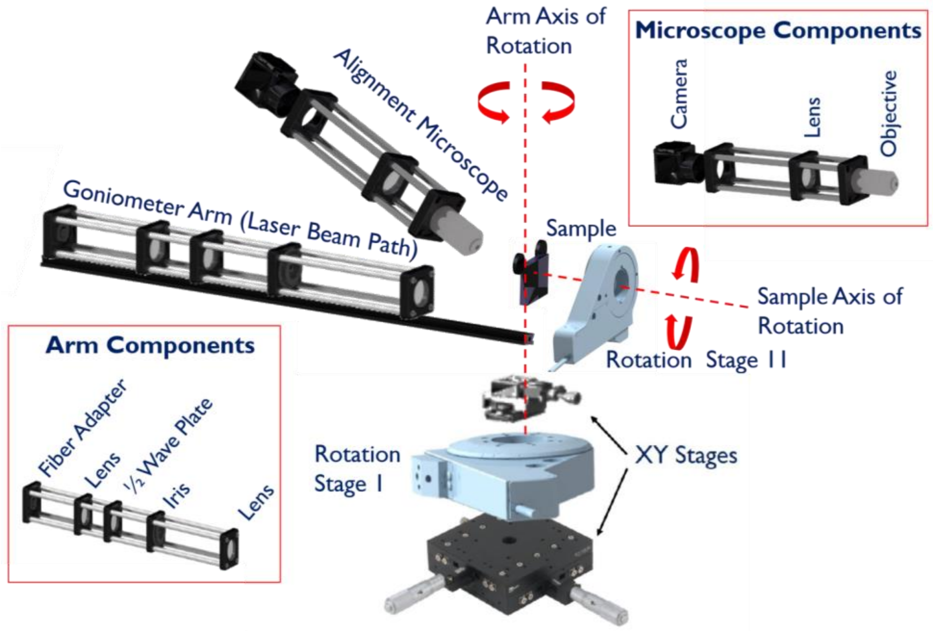

Supplementary Figure 6. Schematic illustration of the angle-resolved photocurrent measurement setup.

In the photocurrent measurements, the polar angle is varied in steps of 1° between ±85°, whereas the measured azimuthal angles range from 0° to 90° in steps of 5°. The remaining two

quadrants of the angle-dependent responsivity maps are then filled up based on the mirror symmetry of the device geometry under study with respect to the *x*-*z* plane. A linear interpolation procedure is also used in the resulting color maps to include additional data points between the measured values of ϕ in steps of 1°. Two maps are measured using this procedure, with the half-wave plate oriented so that (at normal incidence) the input light is linearly polarized perpendicular and parallel to the grating lines, corresponding approximately to *xz* and *yz* polarizations, respectively, for all incident directions. The results are then added up to each other to obtain the full device response. Supplementary Fig. 7 shows a complete set of experimental results for a device providing peak photodetection near θ_p = 30° [the same device of Fig. 3(f) of the main text]. Specifically, panels (a) and (b) in this figure show the measured *xz*- and *yz*-polarized maps, respectively, plotted with the same color scale. Their sum is presented in panel (c). The same maps including the interpolated values are plotted in panels (d)-(f).

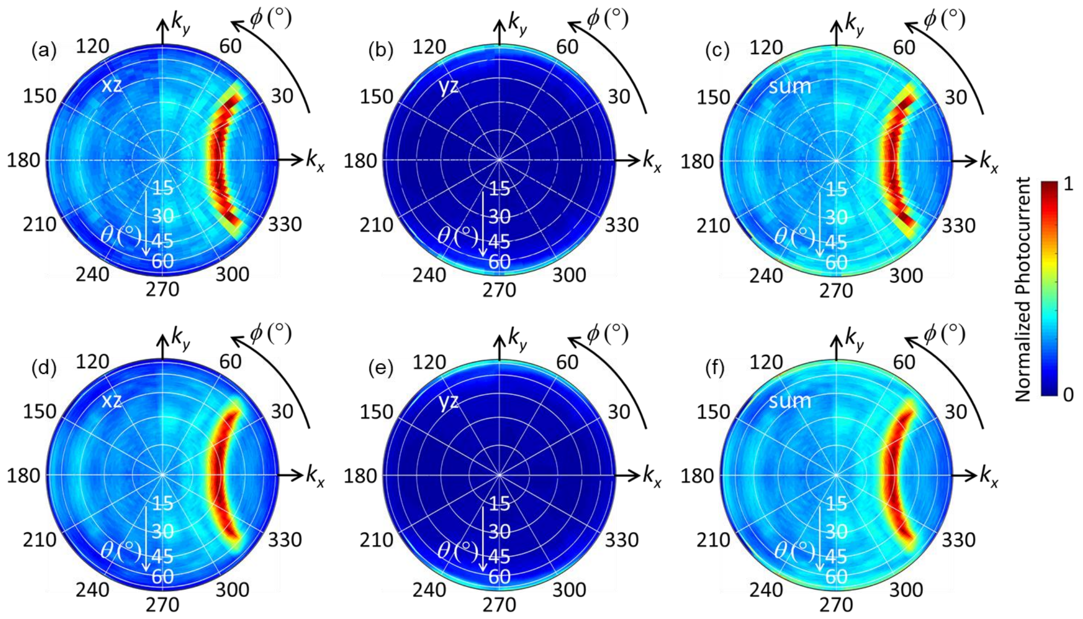

Supplementary Figure 7. Complete set of experimental results for a device providing peak response near θ = 30°. (a), (b), (c) Measured *xz*- and *yz*-polarized maps (plotted with the same color scale), and their sum. (d), (e), (f) Same as (a), (b), (c), respectively, including the interpolated values.

Additional experimental data are plotted in Supplementary Fig. 8, including the full maps of two devices providing peak detection at θ_p = 40° [panel (a)] and 55° [panel (b)], and their horizontal line cuts [panels (c) and (d), respectively].

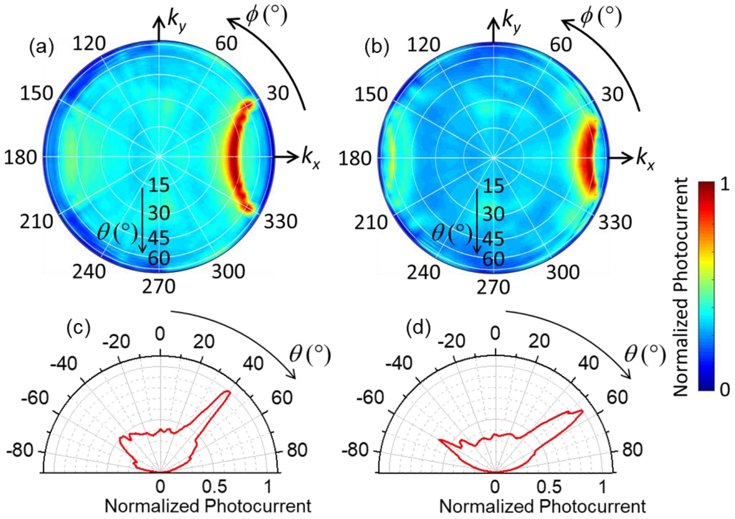

Supplementary Figure 8. Additional experimental results. Measured angular dependence of the photocurrent of two devices based on the structures of Fig. 2 of the main text, providing peak response at θ_p = 40° (a, c) and 55° (b, d). The data shown in each map were summed over *xz* and *yz* polarizations. The grating coupler periods and NP widths, estimated from SEM images, are Λ = 901 and 837 nm and w = 507 and 477 nm for the devices of panels (a) and (b), respectively. Source data for panels (a) and (b) are provided as Source Data files.

Supplementary Note 6. Interpolation methods

The interpolation method used to generate new angular response maps from the calculated or measured ones is summarized in Supplementary Fig. 9. The main idea is to shift and mix two known response maps in order to interpolate a new one whose polar angle of peak detection is in between those of the known maps. As discussed in the main text and illustrated above, the incident directions of high response for different angle-sensitive photodetectors form similar C-shaped regions, centered at different angles. In order to generate the new response map, we shift the C-shaped regions of the known pixel responses to the desired position, and then take their weighted sum. Specifically, each response map is first divided up into two parts, the right half containing the response peak and the left half consisting only of detection background. For the known pixel peaked at lower angle, both parts are shifted away from each other (i.e., to higher angles), as shown in the first row of Supplementary Fig. 9. This shift results in a blank gap in between the two regions, which is filled up by replicating the background values in the middle of the original response (the values along the red dashed line in the figure) across the entire blank area. Similarly, for the known pixel peaked at higher angle, the two halves are shifted towards each other, as shown in the second row of Supplementary Fig. 9, with the alpha blending method [6] used to combine

the overlapping regions. The resulting blank regions near the left and right outer edges of the shifted map are then filled up using a mask multiplied by the original map. Finally, the new response map is obtained from a weighted sum of the two shifted maps plus this additional background correction term, as shown in the third row of Supplementary Fig. 9. The relative weights α and β in the sum are determined based on the respective angular shifts, so that the new map converges to the original ones as the interpolated angle of peak detection approaches those of the original pixels.

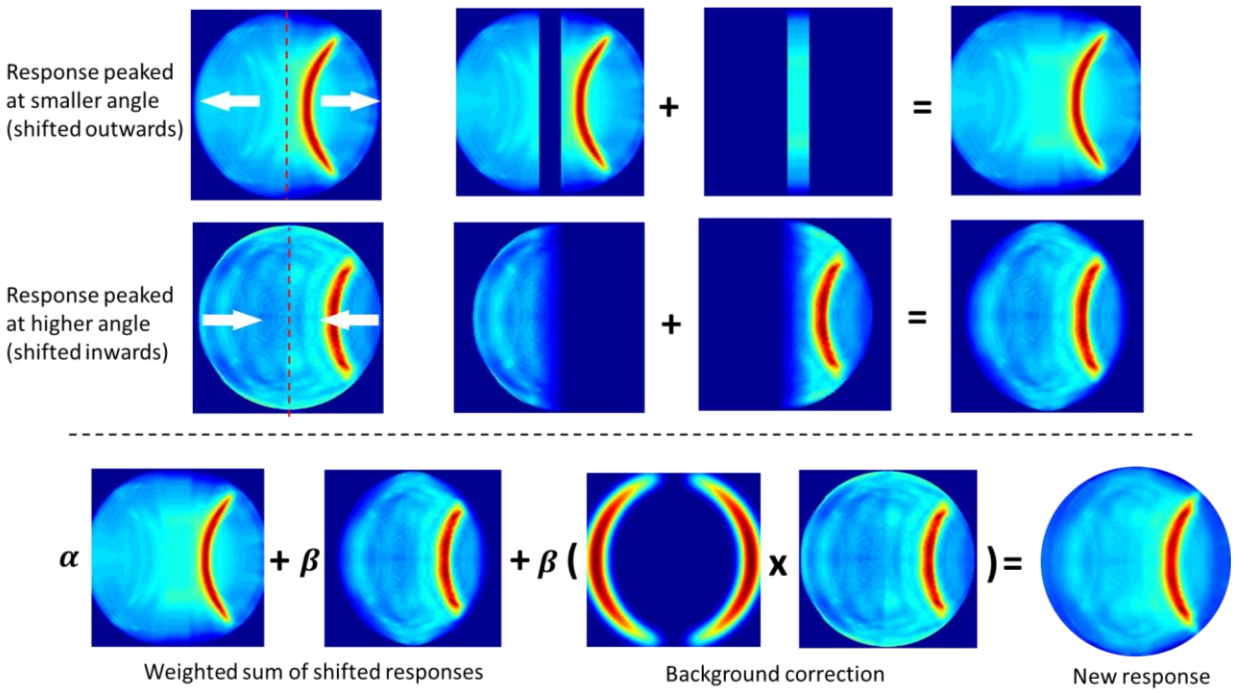

Supplementary Figure 9. Interpolation method used with both simulated and experimental response maps. First row: experimental map peaked at 15°, shifted outwards to simulate a new pixel peaked at a higher angle (22.5°). Second row: experimental map peaked at 30°, similarly shifted inwards to simulate the same new pixel. Third row: interpolated response of the new pixel peaked at 22.5°, obtained from a weighted sum of the two shifted maps plus a background correction term.

With the procedure just described, we have used the numerical (or experimental) maps peaked at, e.g., θ_p = 15° and 30° to generate several new maps with peak angles in between these two values, etc. Representative results are shown in the top two rows of Supplementary Fig. 10. In all cases, no interpolation artifacts are introduced and all the key device characteristics, including angular selectivity, average peak-to-background ratio, and background variations, are comparable to those of the original maps. All the maps produced with this procedure are peaked

at an incident direction with $\phi_p = 0°$, as in all simulated or measured maps. Additional maps peaked at nonzero azimuthal angles were then generated simply by rotating their $\phi_p = 0°$ counterparts.

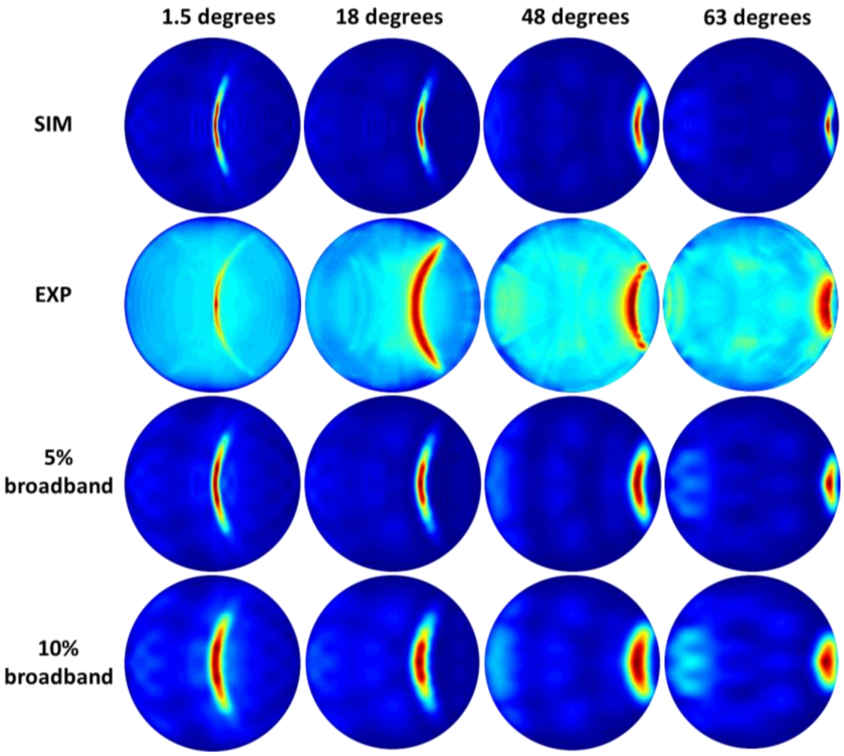

Supplementary Figure 10. Examples of interpolated angular response maps. Top row: results obtained from the calculated maps. Second row: results obtained from the measured maps. Third and fourth row: results obtained from the calculated maps assuming polychromatic illumination with a 5- and 10-% optical bandwidth, respectively.

In order to simulate the response of our devices under polychromatic illumination, the calculated maps are convolved with a 1D Gaussian kernel along k_x (the x component of the incident wavevector). From the diffraction condition discussed in the main text, in the presence of incident broadband light with center wavelength λ_0 and bandwidth $\delta\lambda$, the detection peak is broadened along the k_x direction by the angular amount $\delta\theta_p = \delta\lambda/\lambda_0(n_{SPP} + \sin\theta_p)/\cos\theta_p$, where θ_p is the polar angle of peak detection at λ_0, and n_{SPP} is the plasmonic effective index. The FWHM of the Gaussian kernel for each pixel design is determined from this formula. Examples of simulated broadband response maps are shown in the bottom two rows of Supplementary Fig. 10 for $\delta\lambda/\lambda_0$ = 5 and 10%.

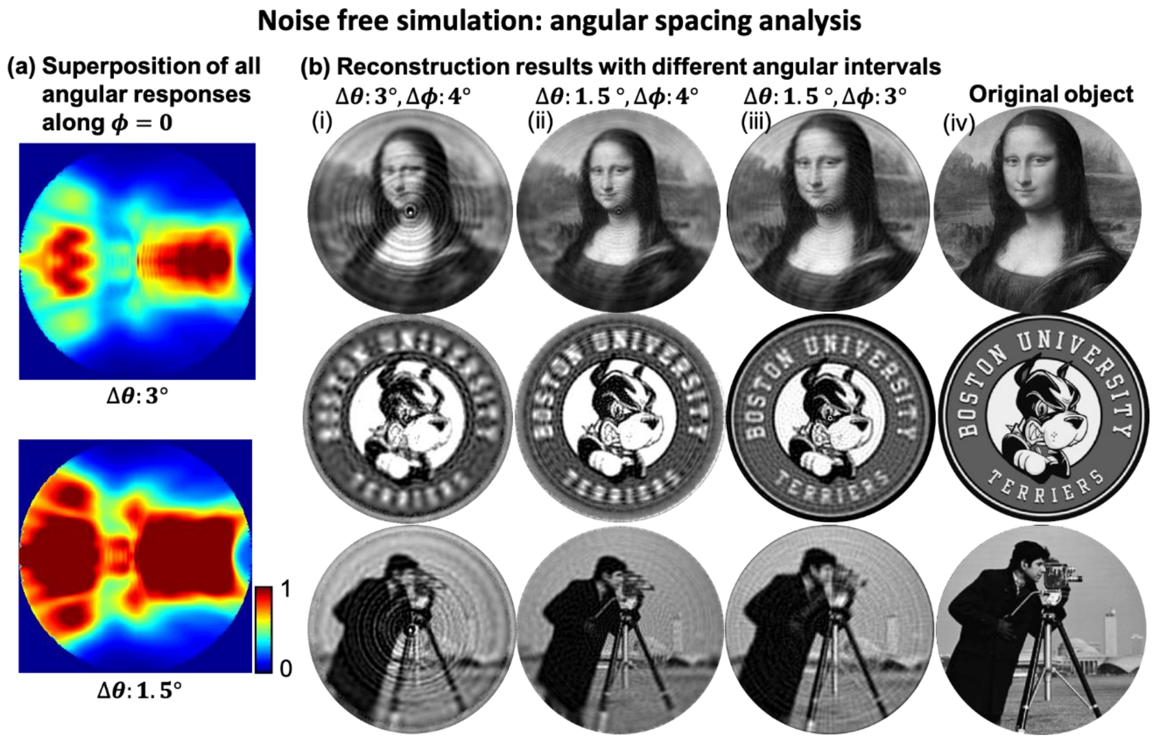

Supplementary Figure 11. Angular-spacing analysis. (a) Superposition of all simulated and interpolated pixel response maps peaked at $\phi_p = 0$ for two different spacings between consecutive values of θ_p, 3° (top) and 1.5° (bottom). The latter choice is found to be small enough to ensure uniform coverage across the desired field of view. (b) Noise-free image reconstruction results under different angular spacing conditions. Large θ_p spacing results in fringe artifacts along the radial direction. Large ϕ_p spacing results in reduced resolution, especially in the high-polar-angle regions of the field of view. The original cameraman image is used with permission from its copyright owner (Massachusetts Institute of Technology).

Supplementary Note 7. Interpixel angular-spacing analysis

Here we investigate how the image reconstruction results are affected by the spacings $\Delta\theta_p$ and $\Delta\phi_p$ between the angles of peak detection of consecutive pixels, which determine the number of pixels needed to cover the full field of view. Supplementary Fig. 11(a) shows the superposition of all simulated and interpolated response maps peaked at $\phi_p = 0°$ and different values of θ_p (equally spaced between 0° and 75°). For the larger spacing $\Delta\theta_p$ considered in this figure (3°), some noticeable gaps along the $\phi_p = 0°$ axis are observed in the superposition (particularly near the center of the field of view), which can produce fringe artifacts in the reconstructed images as illustrated in Supplementary Fig. 11(b)-(i). In contrast, when $\Delta\theta_p$ is decreased to 1.5°, the field of view appears to be fully covered by the combined maps (again along the $\phi_p = 0°$ axis), and the image artifacts are correspondingly removed as shown in Supplementary Fig. 11(b)-(ii). The image

reconstruction quality is also affected by the spacing in ϕ_p. In particular, comparison between columns (ii) and (iii) of Supplementary Fig. 11(b) shows that the image resolution increases with decreasing $\Delta\phi_p$, especially in the high-polar-angle regions of the field of view. Based on these results (which incidentally were produced assuming noise-free measurements for simplicity), we choose $\Delta\theta_p = 1.5°$ and $\Delta\phi_p = 3°$ for all imaging simulations presented in the main text and below.

Supplementary Note 8. Simulation of noisy measurements and additional examples of image reconstruction results

Noisy measurements are simulated by adding white Gaussian noise to the captured photocurrent signal A_{signal} of each pixel, with standard deviation A_{noise} determined from the signal-to-noise ratio (SNR) of the pixel array, i.e.,

$$\mathrm{SNR} = \frac{A_{\mathrm{signal}}}{A_{\mathrm{noise}}}, \qquad \mathrm{SNR_{dB}} = 20\log_{10}\left(\frac{A_{\mathrm{signal}}}{A_{\mathrm{noise}}}\right).$$

In all simulations presented in this work we use three different SNR values, 56, 63, and 73 dB, which can be achieved with standard CMOS technology and different degrees of pixel averaging.

Additional examples of reconstructed images are presented below, for several objects of varying complexity imaged at the three different SNR values just mentioned. In particular, Supplementary Fig. 12 shows reconstruction results based on both simulated and measured angular response maps at the target design wavelength of 1550 nm. Supplementary Fig. 13 shows reconstruction results for polychromatic illumination with $\delta\lambda/\lambda_0$ = 5 and 10%.

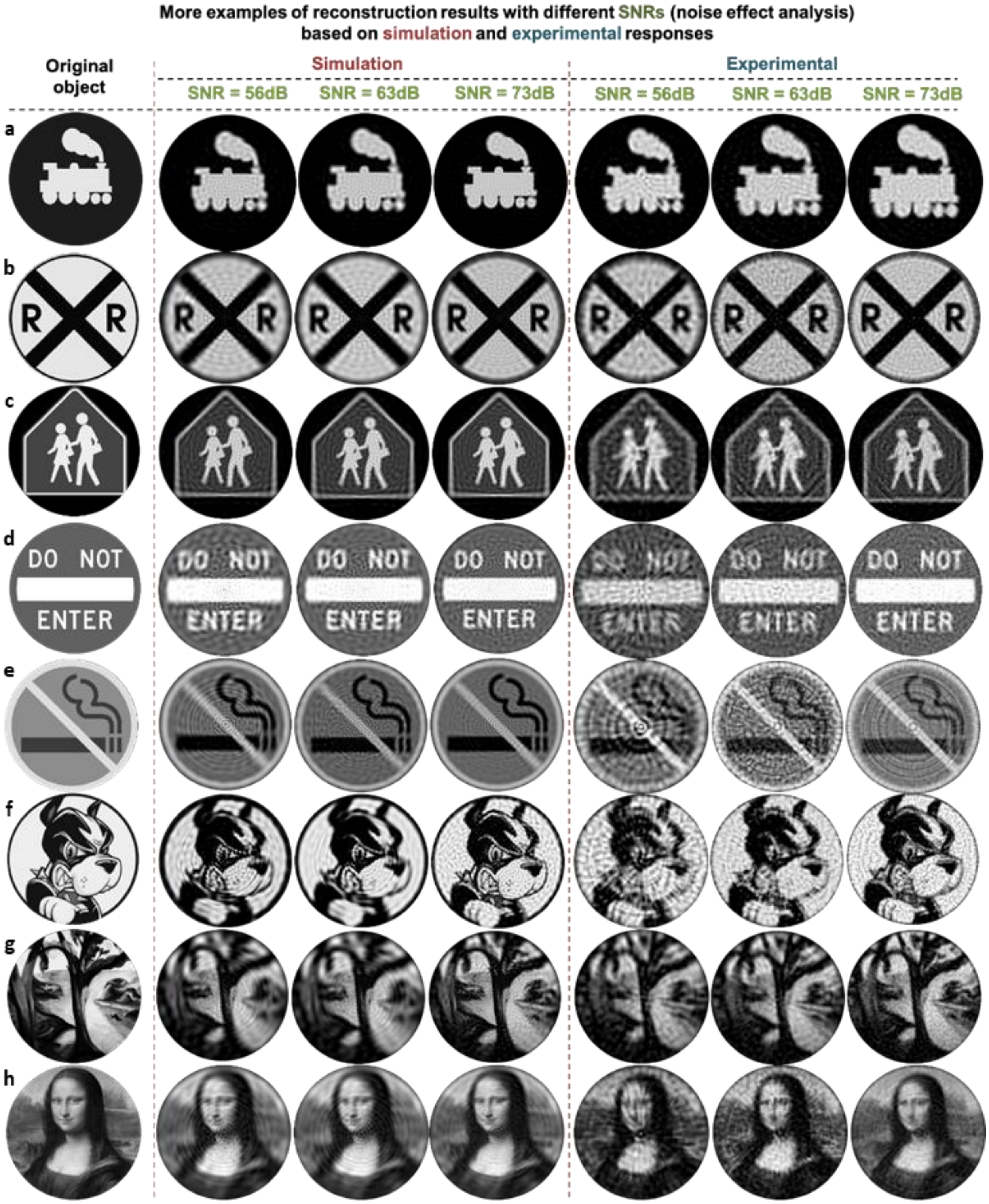

Supplementary Figure 12. Additional examples of reconstruction results at λ_0 = 1550 nm for both simulated and experimental devices under three different SNRs. The original object of row **a** (train crossing sign) is Crown copyright. The original object of row **g** (tree image) is used with permission from the author (Susan Cohen Thompson, thompsonartstudio.com).

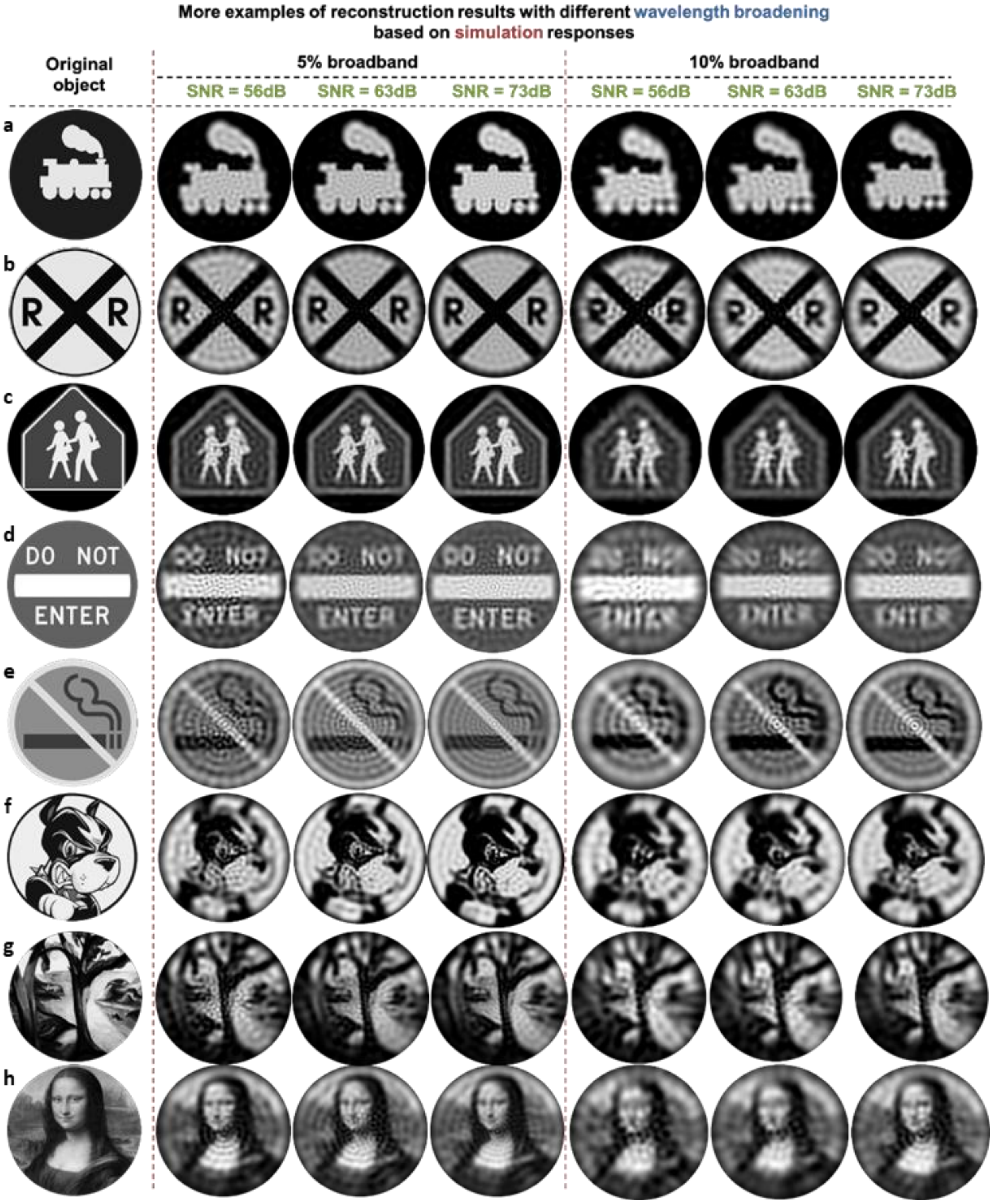

Supplementary Figure 13. Additional examples of broadband reconstruction results for two different bandwidths under three different SNRs. The original object of row **a** (train crossing sign) is Crown copyright. The original object of row **g** (tree image) is used with permission from the author (Susan Cohen Thompson, thompsonartstudio.com).

Supplementary References

1. Chu, Y. & Crozier, K. B. Experimental study of the interaction between localized and propagating surface plasmons. *Opt. Lett.* **34**, 244-246 (2009).
2. Ghoshal, A., Divliansky, I. & Kik, P. G. Experimental observation of mode-selective anticrossing in surface-plasmon-coupled metal nanoparticle arrays. *Appl. Phys. Lett.* **94**, 171108 (2009).
3. Yu N. & Capasso, F. Flat optics with designer metasurfaces. *Nature Mater.* **13**, 139-150 (2014).
4. Ding, F., Pors, A. & Bozhevolnyi, S. I. Gradient metasurfaces: a review of fundamentals and applications. *Rep. Prog. Phys.* **81**, 026401 (2018).
5. Pors, A., Nielsen, M. G., Bernardin, T., Weeber, J.-C. & Bozhevolnyi, S. I. Efficient unidirectional polarization-controlled excitation of surface plasmon polaritons. *Light: Sci. Appl.* **3**, e197 (2014).
6. Porter, T. & Duff, T. Compositing digital images. *ACM SIGGRAPH Computer Graphics* **18**, 253–259 (1984).
