# Supplementary material for: Plasmonic ommatidia for lensless compound-eye vision
Source: Nat Commun. 2020 Apr 2;11:1637. doi: 10.1038/s41467-020-15460-0 (PMC7118074; doi:10.1038/s41467-020-15460-0)
Supplement: Supplementary file 3 — Description of Additional Supplementary Files [file 41467_2020_15460_MOESM3_ESM.pdf]

Description of Additional Supplementary Files

File Name: Supplementary Movie 1
Description: Time-dependent optical-field distribution near the metasurface of the $\theta_p$=45° device, computed by 2D FDTD simulations with PML boundary conditions. The device is illuminated with a *p*-polarized pulsed Gaussian beam with 80-fs pulse-width and 90-nm spectral bandwidth (centered at 1550 nm) incident along the target direction of peak photodetection. The movie shows the coupling of the incident light to SPPs propagating towards the slits, and the subsequent scattering of these SPPs by the slits into the substrate. It should be noted that the overall transmission across the metasurface in these simulations is somewhat limited by the finite convergence angle and bandwidth of the input wave.

File Name: Supplementary Movie 2
Description: Same as Supplementary Movie 1, except that the input light is incident at the equal and opposite angle $\theta$= –45° .The movie shows the coupling of the incident light to SPPs propagating towards the grating reflector, and the subsequent scattering of these SPPs by the reflector into the free space above the metasurface.
